# A Survey Study of Veterinary Student Opinions and Knowledge about Pet Reptiles and Their Welfare †

**DOI:** 10.3390/ani11113185

**Published:** 2021-11-08

**Authors:** Mario Ostović, Ivana Sabolek, Aneta Piplica, Ivona Žura Žaja, Sven Menčik, Srebrenka Nejedli, Željka Mesić

**Affiliations:** 1 Department of Animal Hygiene, Behaviour and Welfare, Faculty of Veterinary Medicine, University of Zagreb, Heinzelova 55, 10000 Zagreb, Croatia; isabolek@vef.unizg.hr; 2 Department of Animal Breeding and Livestock Production, Faculty of Veterinary Medicine, University of Zagreb, Heinzelova 55, 10000 Zagreb, Croatia; apiplica@vef.unizg.hr (A.P.); smencik@vef.unizg.hr (S.M.); 3 Department of Physiology and Radiobiology, Faculty of Veterinary Medicine, University of Zagreb, Heinzelova 55, 10000 Zagreb, Croatia; izzaja@vef.unizg.hr; 4 Department of Anatomy, Histology and Embryology, Faculty of Veterinary Medicine, University of Zagreb, Heinzelova 55, 10000 Zagreb, Croatia; snejedli@vef.unizg.hr; 5 Department of Marketing in Agriculture, Faculty of Agriculture, University of Zagreb, Svetošimunska Cesta 25, 10000 Zagreb, Croatia; zmesic@agr.hr

**Keywords:** veterinary students, survey, exotic pets, reptiles, animal welfare

## Abstract

**Simple Summary:**

Very few studies have investigated veterinary perception of reptiles kept as pets. Using 5-point Likert scale questions, this study assessed opinions of veterinary students in Croatia about pet turtles, lizards and snakes, and their welfare, including student self-reported knowledge about pet reptiles. Most of the students’ responses were neutral from the very beginning to the end of their study, emphasising the need for introducing additional veterinary education on pet reptiles, with implications for the welfare of these pets, health and safety of humans and other animals, and environmental protection.

**Abstract:**

Exotic pet medicine is rapidly evolving, with reptiles becoming increasingly popular pet animals. Yet, there are only a few literature reports on veterinary perception of reptiles kept as pets. The aim of the study was to assess opinions and knowledge of the Croatian veterinarians-to-be about pet reptiles and their welfare. The questionnaire survey was conducted in the academic year 2019–2020 and included students of all six years of the integrated undergraduate and graduate study at the Faculty of Veterinary Medicine, University of Zagreb. First-year students were surveyed twice, before and after having attended the compulsory course on animal welfare. Questionnaire statements were 5-point Likert scale questions, requiring the students to express their opinions about turtles, lizards and snakes as pets, issues related to their welfare, risks they pose to the health and safety of humans, other animals and the environment, and their self-reported knowledge about pet reptiles. Although expressing higher opinions after having attended the course on animal welfare, first-year student responses remained neutral to most of the statements. Such a trend continued until the end of the study. Student responses revealed that they were uncertain about their knowledge of reptiles as pets, considering different educational areas observed. Study results emphasised the need of alterations in veterinary curriculum and additional student education in reptile medicine. The results obtained have broad implications involving not only the welfare of pet reptiles in clinical practice and elsewhere but also the health and safety of humans and other animals, as well as environmental protection.

## 1. Introduction

Veterinarians are expected to have a leading role in ensuring and promoting animal welfare [1] but their perception of animal welfare may differ depending on animal observed. For instance, veterinary attitudes and opinions, including those of veterinary students in Croatia [2,3] have been shown to be more positive toward pet dogs and cats than toward farm animals [4,5,6].

Exotic pet medicine is a rapidly growing discipline of veterinary medicine [7,8]. However, the ever-increasing number and diversity of pet species undoubtedly present new challenges and efforts for veterinary professionals [3], with the health and welfare of exotic pets depending on the knowledge and understanding of their environmental, nutritional and behavioural needs [9]. Veterinarians are very familiar with health issues encountered in domestic animals and therefore are highly competent to provide proper advice and guidelines on keeping and care of these animals, unlike exotic, nondomesticated species about which it is quite difficult to get competent advice due to the relatively small number of veterinarians specialised in these animal species [10,11]. A study by Siğirci et al. [8] conducted in Istanbul, Turkey, revealed that 90% of small pet practitioners consider themselves to have received inadequate education in exotic pet animal practice during their undergraduate study. The veterinarians reported appropriate knowledge and experience regarding birds (65%) but not turtles, other reptiles and fish.

The class Reptilia comprises nearly 11,000 species [12,13], many of which are becoming ever more popular pets, with 9.1 million reptiles kept as pets in Europe alone [14]. However, keeping and trading reptiles as pets, which accounts for a considerable portion of the live animal trade [15], raises ever more issues mostly related to their welfare, public health and safety (e.g., reptile-associated salmonellosis), species preservation, invasiveness and environmental degradation [11,16]. Florida is one of the best-known examples with the highest number of established, i.e., reproducing non-native herpetofaunal species in the world, with the pet trade being the most common invasion pathway [17].

According to Toland et al. [18], 75% of pet reptiles die within a year of acquisition, although other authors report considerably lower figures [15]. Welfare issues in pet reptiles remain very disturbing at all points in the chain from capture/breeding to sales/housing [11,19]. Animals sold as captive-bred may in fact be wild-caught [11]. It is estimated that 25% of exotic pet trade worldwide is illegal [11]. Unlike other vertebrates such as mammals or birds, reptiles frequently evoke aversion in humans, which can preclude their protection [12].

Exotic pet suitability, including reptile species, has been widely disputed recently among veterinarians as well [19,20]. A study by Whitehead et al. [21] revealed that most veterinarians in the United Kingdom believed that welfare needs of pet reptiles were not met properly. The predominant opinion was that wild-caught reptiles should not be kept as pets, whereas their opinions about captive-bred reptiles differed, yet many veterinarians considered keeping them as pets unacceptable.

We embarked upon this study to assess the opinions and knowledge of Croatian veterinarians-to-be about reptiles kept as pets and their welfare.

## 2. Materials and Methods

This questionnaire survey was performed at the only Faculty of Veterinary Medicine in Croatia, at the University of Zagreb, in the winter term of the academic year 2019–2020. The written questionnaire was answered by students of all six years of the integrated undergraduate and graduate study. First-year students answered the questionnaire twice, before and after having attended a compulsory course on animal welfare, yielding two overall response rates of 88% (*n* = 596) and 87% (*n* = 589), respectively. Students of all study years had the same curriculum, having acquired knowledge and practice related to exotic pets throughout their study, with few elective courses focusing specifically on reptiles. Prior to filling out the questionnaire, students were informed on the objectives of the research and that the results obtained would be used for scientific and educational purposes. Student participation in the research was voluntary and anonymous.

The questionnaire consisted of two parts (as shown in the Supplementary Materials). The first group of questions referred to student demographic data, i.e., gender, age, early environment, secondary education, owning or keeping pets including exotic pets (i.e., any species other than dogs and cats), study year, and on the preferred, i.e., chosen study track in the 10th semester. The second part of the questionnaire included 15 statements in the form of 5-point Likert scale questions (1—totally disagree to 5—totally agree), designed to enable assessment of student opinions about pet reptiles, i.e., turtles (Chelonians), lizards and snakes, and their welfare, and student self-reported knowledge about these pets. The questionnaire was pretested by three experts in the field and a presurvey was carried out in 10% of students in each year. Reliability (α) of the questionnaire was 0.845.

The data collected in the study were analysed by use of IBM SPSS Statistics v. 21.0 (IBM Corp., Armonk, NY, USA, 2012). Frequencies of student responses were determined by univariate analysis. Student opinions and knowledge were estimated by calculating mean responses to particular statements. On calculating total mean responses to statements (mean value of all study years), as well as total responses referring to student data, responses given by first-year students after having attended the course on animal welfare were taken in consideration. Differences in student responses among study years and differences in total mean responses were analysed by Kruskal–Wallis test and Mann–Whitney *U*-test, whereas differences between first-year student responses before and after having attended the course on animal welfare were analysed by Wilcoxon signed-rank test. Difference at the level of *p* < 0.05 was considered significant in all tests.

## 3. Results

Demographic data showed 78.8% of all respondents to be female; 54% belong to the 18–21 age group; 69.9% had been grown up in urban setting; 88.8% had finished high school; 97.3% owned or kept pets including exotic pets (51.6%); and the highest proportion of students (40.6%) strove for a career in pet medicine.

**Figure 1:**
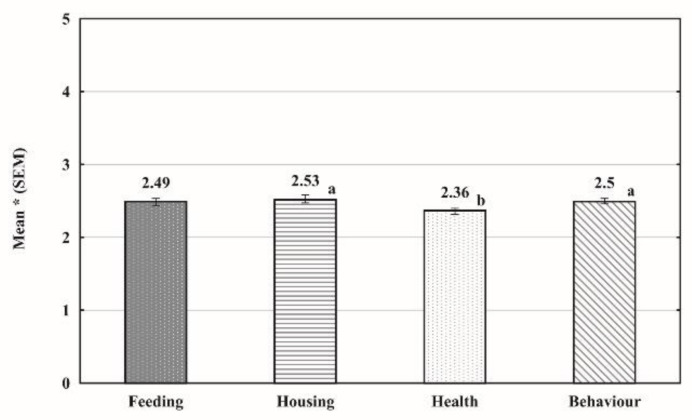
Total mean responses of veterinary students (*n* = 589) on their self-reported knowledge about pet reptile feeding, housing, health and behaviour. * 1—totally disagree; 5—totally agree; ^a,b^—values marked with different letters differ significantly (*p* < 0.05).

Total mean student responses to questionnaire statements and respective significant differences are shown in Table 1 and Figure 1. In order to make the presentation of the results according to study years more convenient, significant differences in mean responses between first-year and sixth-year students, and between first-year students before and after having attended the course on animal welfare are shown in Table 2, Table 3, Table 4 and Table 5. Mean responses of students of all study years are presented in the Supplementary Materials (Tables S1–S4).

As shown in Table 1, students totally agreed with the statements that biological functioning and natural living were important welfare components of pet turtles, lizards and snakes, whereas they were less certain about the importance of emotional states for their welfare. Students were indecisive whether these reptiles were capable of thinking and feeling emotions, whether it was acceptable to keep them as pets, and whether their owners were adequately informed about them prior to acquisition, questioning their welfare. Students considered that turtles and lizards did not pose a health and safety risk for humans and other animals, but neither disagreed nor agreed about snakes. None of these pet animals was described as an environmental risk. Students provided neutral responses to most of these statements; yet, considered turtles significantly more (*p* < 0.05) capable of feeling emotions, with emotional states being significantly more (*p* < 0.05) important for their welfare, when compared with lizards and snakes. In addition, students considered snakes to be significantly less (*p* < 0.05) acceptable as pet animals while posing a significantly greater (*p* < 0.05) environmental risk in comparison to turtles and lizards. Concerning health and safety risk for humans and other animals, students ranked the study reptiles as follows: snakes > lizards > turtles (*p* < 0.05 for all).

As shown in Figure 1, students did not agree or were indecisive concerning their self-reported knowledge about feeding, housing, health and behaviour of pet reptiles, with special reference to health relative to housing and behaviour (*p* < 0.05 both).

To statements presented in Table 2, Table 3 and Table 4, first-year students mostly gave neutral responses before having attended the course on animal welfare. After this course, they provided significantly higher (*p* < 0.05) mean responses, in particular to statements on the issues of pet lizard welfare (Table 3) and the risk posed by pet turtles for health and safety of humans and other animals, and for the environment (Table 2). However, student responses still were mostly neutral, including significantly less (*p* < 0.05) mean responses concerning level of information acquired by the owners of pet turtles, lizards and snakes prior to owning them (Table 2, Table 3 and Table 4). The majority of sixth-year student mean responses were neutral, with less significant differences from mean responses given by first-year students after having attended the course on animal welfare than between mean responses of first-year students given before and after having attended the course on animal welfare (Table 2, Table 3 and Table 4). Accordingly, student responses to most of the statements were neutral throughout the six-year study (Tables S1–S4). There were no significant differences in student responses among particular study years concerning statements related to compromised welfare of pet turtles and snakes, acceptability of turtles and lizards as pets, level of information of owners about pet turtles, risk posed by pet lizards and snakes for health and safety of other animals and environment, and risk of pet lizards for health and safety of humans. There were no significant differences in student responses among particular study years concerning statements related to their self-reported knowledge about feeding, housing, health and behaviour of pet reptiles either, including differences between responses given by sixth-year students and first-year students before and after having attended the course on animal welfare. The only significant difference (*p* < 0.05) was recorded between the mean responses of first-year students before and after having attended the course on animal welfare regarding their self-reported knowledge about pet reptile health (Table 5).

## 4. Discussion

Study results revealed veterinary students to totally agree with statements that biological functions and natural living were important for pet reptile welfare, while expressing low level of agreement concerning the role of emotional states for their welfare. Students were not sure about reptile cognition and sentience; yet, feelings were significantly more frequently attributed to turtles than lizards and snakes, which could be explained by turtles being more commonly kept as pets than other reptiles.

Cognition refers to mental activities or processes by which animals perceive, process and store information [22]. Sentience, on the other hand, refers to an animal’s capacity to feel and being aware of various states and sensations such as pleasure and suffering [22]. Students are expected at least to take pain into consideration as a feeling, knowing that pain is experienced by all vertebrates [23]. Although signs of pain and suffering in reptiles can be challenging to recognise [24,25,26], the knowledge about cognition and capacity for pain and other feelings in reptiles is clear enough to justify the arguments for their protection. This has direct implications for managing captive reptiles, because better understanding of their cognition and sentience is crucial to ensure the best quality of life for these animals [13,26,27].

Although student responses to statements related to acceptability of turtles, lizards and snakes as pets differed significantly, students were indecisive on whether reptiles are acceptable as pets. Students were also indecisive concerning the owner level of information about reptiles as pets and their needs prior to acquiring them, thus considering their welfare potentially compromised.

Keeping reptiles as pets raises numerous welfare issues. Nutritional metabolic bone disease associated with calcium deficiency is one of the most common pathological and painful states in pet reptiles. Many reptiles develop disease as a result of hyperthermia, including thermal burns, or chronic hypothermia due to inappropriate heating [25,28]. Other issues include trauma due to attempted escape, inappropriate handling, limited movement in enclosures of inadequate size, etc. [19]. Warwick et al. [29] report that captive environments for snakes usually include small enclosures, the dimensions of which prevent them from acquiring straight-line body posture; therefore, future policies of keeping snakes should take their greater spatial need into consideration. With time, many reptiles become too big or too expensive to keep as pets; in addition to this, their longevity should also be considered. Corn snakes and green iguanas can live for 20 or more years, and red-eared turtles for more than 40 years. Such species can outlive their owners, or the owners can lose the interest or ability to care for them, resulting in taking the animal to a shelter, to another owner, or just being released into the wildlife [28].

Exotic alien species may displace native species through predation, hybridization, pathogen transmission, or competition for resources, threatening non-native ecosystems, natural resources, and even human health [28,30]. Burmese python in Florida is just one of many examples of invasive alien species being introduced into non-native environments. For instance, McCleery et al. [31] found this species (Burmese python) to account for 77% of all marsh rabbit mortalities within 11 months of their translocation to the Everglades National Park. Our students are either not familiar with the potential invasiveness of pet reptiles or do not associate it with the reptiles kept as pets in Croatia, or they just did not think of it when questioned, but they consider none of the pet reptiles observed to pose a risk to the environment, although responses to this statement yielded significant differences among particular reptiles. In 2017, the European Commission published a brochure on 49 invasive alien plant and animal species of concern in the European Union [32]. Seventeen species from the list have been recorded in Croatia, including pond slider and red-eared turtle as its most popular subspecies in pet trade [33]. It has been estimated that 50 million individual animals of this species (pond slider) have been imported into Europe for the pet trade over time. Many of these animals have escaped or have been released to wildlife. Currently, the species is present in 22 EU member countries [32]. The potential invasiveness of non-native reptiles as pets for native species and ecosystems may not be an obligatory topic covered by the veterinary curriculum, however, students could have been familiarised with this issue via other sources such as media (e.g., Internet, television).

Although the study results showed pet snakes to pose a significantly higher risk for the health and safety of humans and other animals, in comparison with both lizards and turtles, and also lizards compared to turtles, students were indecisive about whether snakes pose such a risk, and considered lizards and turtles not to pose a risk. It should be borne in mind that these are still wild animals with intact defensive and aggressive behaviour, along with robust physical attributes [34], which can not only cause injuries or poisoning, but can also be fatal for both humans and other animals. Besides this, students should be aware that pet reptiles can also threaten the health of humans and other animals by transmission of diseases. A common example of pet-linked zoonoses is reptile-associated salmonellosis. Mermin et al. [35] report reptile and amphibian exposure to be associated with approximately 74,000 human *Salmonella* infections annually in the United States. Children are at the highest risk of reptile-related salmonellosis. A study conducted in the United Kingdom showed 27% of *Salmonella* cases in children aged <5 years to be associated with reptiles [36]. A German study also demonstrated that reptiles, bearded dragons in particular, shed various *Salmonella* serovars, including those isolated in infected children from study households [37]. Lukac et al. [38] found 13% of reptiles belonging to private owners in Croatia or the Zagreb ZOO to be positive for *Salmonella*, including 48% of lizards, 8.9% of snakes and 3.8% of turtles. The authors conclude that the prevalence of *Salmonella* in captive reptiles in Croatia is considerable and that these animals can harbour serovars which generally are not seen in veterinary or human microbiological routine, calling for due attention to this issue in the prevention and diagnosis of human reptile-transmitted infections. Therefore, good hygiene practice related to husbandry is of utmost importance for the prevention of reptile-associated salmonellosis. Its prevention should be based on proper information and education, with a primary role of veterinary professionals in managing this task [39]. Our students considered that they had not acquired due knowledge about feeding, housing, health and behaviour of pet reptiles through their education, or were indecisive on the issue; this in particular held true for pet reptile health when compared with their housing and behaviour. These results are consistent with those reported by Siğirci et al. [8] in a study on veterinary practitioners in Turkey.

We also analysed student responses according to study years, as well as before and after the course on animal welfare in first-year students, in order to assess the extent to which ongoing education would provoke changes in their opinions and knowledge during the study. Although the greatest proportion of higher responses was recorded after the course on animal welfare in first-year students, they generally remained indecisive, maintaining such a trend throughout the study. Student responses to most statements yielded no significant differences between sixth-year students and first-year students after having attended the course on animal welfare. A great proportion of statements showed no significant differences in responses among other study years either, including responses to statements on their self-reported knowledge about feeding, housing, health and behaviour of pet reptiles. There were no differences in student responses to statements on their self-reported knowledge about pet reptiles between sixth-year students and first-year students at the very beginning of the study, suggesting deficient student education regarding pet reptiles.

A study by Vučinić et al. [40] revealed that 40% of reptile owners from Bosnia and Herzegovina, Macedonia, Montenegro and Serbia had never contacted or had any experience with veterinarians; 58% contacted or visited veterinarians for some health problems in their pets, 14% contacted veterinarians for advice about keeping these pets, and only 6% of owners had taken their pet reptiles to veterinarians for preventive examination; 47% of pet reptile owners expressed satisfaction with veterinary service. The authors believe that their results could serve as a basis for adopting legislation on pet reptile ownership, as well as for monitoring of subsequent changes in interest for these animals as pets. These results also point to the need of higher veterinarian enthusiasm to educate reptile owners, and to the necessity of veterinary education modification. This is in accordance with the results of the present study.

As student responses to most of the questionnaire statements were neutral through-out the six-year study period, the impact of demographic data on their responses was not investigated. The limitations of the study could be related to its cross-sectional rather than longitudinal design. A longitudinal design would involve surveying the same student population throughout the study period. Moreover, this study assumed general insight into student perception of pet reptiles, not analysing the impact of elective teaching on their opinions and knowledge about these pets and their welfare, which should be taken in consideration in future studies. Further studies should also investigate student opinions and knowledge about other exotic pets (e.g., exotic mammal pets) and their welfare.

## 5. Conclusions

Based on the study results revealing mostly neutral student responses to statements related to pet reptiles and their welfare, including student self-reported knowledge about these pets throughout their study, we find that efforts should be invested to upgrade veterinary curriculum and to introduce additional education of Croatian veterinarians-to-be in reptile medicine. Besides reptiles in clinical practice and elsewhere, this issue has implications for the health and safety of humans and other animals, as well as for environmental protection. These study results can help in upgrading veterinary student knowledge about pet reptiles, thus increasing their awareness of these pet animals and contributing to veterinary perception of pet reptiles and their welfare both in Croatia and worldwide.

## Figures and Tables

**Table 1:** Total mean responses of veterinary students (*n* = 589) to statements related to pet turtles, lizards and snakes.

Statement	Turtles	Lizards	Snakes
	Mean * (SEM)		
These reptiles are capable of thinking	3.11 (0.04)	3.04 (0.04)	3.03 (0.04)
These reptiles are capable of feeling emotions	3.06 ^a^ (0.04)	2.81 ^b^ (0.04)	2.83 ^b^ (0.05)
Biological functioning is important for their welfare	4.71 (0.03)	4.69 (0.03)	4.68 (0.03)
Emotional states are important for their welfare	3.75 ^a^ (0.05)	3.58 ^b^ (0.05)	3.49 ^b^ (0.06)
Natural living is important for their welfare	4.58 (0.04)	4.57 (0.04)	4.53 (0.04)
It is acceptable to keep these reptiles as pets	3.18 ^a^ (0.05)	3.12 ^a^ (0.05)	2.97 ^b^ (0.05)
Owners are properly informed prior to acquisition	3.06 (0.05)	3.14 (0.05)	3.17 (0.05)
The welfare of these pet reptiles is compromised	3.50 (0.04)	3.51 (0.04)	3.51 (0.05)
These pet reptiles pose risk for health and safety of humans	2.09 ^a^ (0.04)	2.30 ^b^ (0.04)	2.83 ^c^ (0.05)
These pet reptiles pose risk for health and safety of other animals	2.12 ^a^ (0.05)	2.33 ^b^ (0.05)	2.76 ^c^ (0.05)
These pet reptiles pose risk for environment	2.14 ^a^ (0.05)	2.13 ^a^ (0.05)	2.31 ^b^ (0.05)

* 1—totally disagree; 5—totally agree; ^a,b,c^—values in the same row marked with different letters differ significantly (*p* < 0.05).

**Table 2:** Mean responses of first-year and sixth-year veterinary students to statements related to pet turtles.

Statement	Study Year		
	First ^A^ (*n* = 130)	First ^B^ (*n* = 123)	Sixth (*n* = 95)
	Mean * (SEM)		
These reptiles are capable of thinking	2.82 ^a^ (0.07)	3.24 ^b^ (0.08)	3.04 (0.10)
These reptiles are capable of feeling emotions	2.65 ^a^ (0.09)	3.11 ^b^ (0.08)	2.91 (0.11)
Biological functioning is important for their welfare	4.44 ^a^ (0.08)	4.61 ^a^ (0.06)	4.78 ^b^ (0.07)
Emotional states are important for their welfare	3.22 ^a^ (0.10)	3.84 ^b^ (0.09)	3.48 (0.14)
Natural living is important for their welfare	3.70 ^a^ (0.10)	4.21 ^b^ (0.10)	4.71 ^c^ (0.08)
It is acceptable to keep these reptiles as pets	3.09 (0.11)	3.13 (0.11)	3.33 (0.12)
Owners are properly informed prior to acquisition	3.39 ^a^ (0.12)	2.99 ^b^ (0.11)	3.06 (0.13)
The welfare of these pet reptiles is compromised	3.37 (0.09)	3.47 (0.08)	3.47 (0.10)
These pet reptiles pose risk for health and safety of humans	1.86 ^a^ (0.08)	2.24 ^b^ (0.10)	2.37 ^b^ (0.12)
These pet reptiles pose risk for health and safety of other animals	1.99 ^a^ (0.09)	2.45 ^b^ (0.11)	2.45 ^b^ (0.13)
These pet reptiles pose risk for environment	1.99 ^a^ (0.09)	2.43 ^b^ (0.11)	2.28 (0.13)

^A^—answered before attending the course on animal welfare; ^B^—answered after the course; * 1—totally disagree; 5—totally agree; ^a,b,c^—values in the same row marked with different letters differ significantly (*p* < 0.05).

**Table 3:** Mean responses of first-year and sixth-year veterinary students to statements related to pet lizards.

Statement	Study Year		
	First ^A^ (*n* = 130)	First ^B^ (*n* = 123)	Sixth (*n* = 95)
	Mean * (SEM)		
These reptiles are capable of thinking	2.72 ^a^ (0.08)	3.17 ^b^ (0.09)	2.90 ^a^ (0.10)
These reptiles are capable of feeling emotions	2.37 ^a^ (0.09)	2.87 ^b^ (0.08)	2.57 ^a^ (0.11)
Biological functioning is important for their welfare	4.28 ^a^ (0.10)	4.61 ^b^ (0.07)	4.75 ^c^ (0.07)
Emotional states are important for their welfare	2.91 ^a^ (0.10)	3.44 ^b^ (0.12)	3.33 ^b^ (0.14)
Natural living is important for their welfare	3.52 ^a^ (0.11)	4.25 ^b^ (0.10)	4.68 ^c^ (0.08)
It is acceptable to keep these reptiles as pets	2.82 (0.11)	2.92 (0.11)	3.22 (0.13)
Owners are properly informed prior to acquisition	3.59 ^a^ (0.12)	3.08 ^b^ (0.12)	3.17 ^b^ (0.13)
The welfare of these pet reptiles is compromised	3.28 ^a^ (0.08)	3.61 ^b^ (0.09)	3.40 (0.11)
These pet reptiles pose risk for health and safety of humans	2.23 (0.09)	2.41 (0.09)	2.44 (0.12)
These pet reptiles pose risk for health and safety of other animals	2.31 (0.09)	2.51 (0.10)	2.53 (0.13)
These pet reptiles pose risk for environment	2.15 (0.10)	2.16 (0.09)	2.33 (0.13)

^A^—answered before attending the course on animal welfare; ^B^—answered after the course; * 1—totally disagree; 5—totally agree; ^a,b,c^—values in the same row marked with different letters differ significantly (*p* < 0.05).

**Table 4:** Mean responses of first-year and sixth-year veterinary students to statements related to pet snakes.

Statement	Study Year		
	First ^A^ (*n* = 130)	First ^B^ (*n* = 123)	Sixth (*n* = 95)
	Mean * (SEM)		
These reptiles are capable of thinking	2.88 (0.09)	3.11 ^a^ (0.09)	2.80 ^b^ (0.11)
These reptiles are capable of feeling emotions	2.31 ^a^ (0.09)	2.83 ^b^ (0.10)	2.52 ^a^ (0.11)
Biological functioning is important for their welfare	4.23 ^a^ (0.10)	4.52 ^a^ (0.08)	4.71 ^b^ (0.08)
Emotional states are important for their welfare	2.75 ^a^ (0.11)	3.32 ^b^ (0.12)	3.31 ^b^ (0.14)
Natural living is important for their welfare	3.66 ^a^ (0.11)	4.13 ^b^ (0.11)	4.66 ^c^ (0.08)
It is acceptable to keep these reptiles as pets	2.70 ^a^ (0.11)	2.69 ^a^ (0.11)	3.11 ^b^ (0.13)
Owners are properly informed prior to acquisition	3.59 ^a^ (0.12)	2.99 ^b^ (0.12)	3.22 ^b^ (0.14)
The welfare of these pet reptiles is compromised	3.38 (0.09)	3.57 (0.09)	3.38 (0.11)
These pet reptiles pose risk for health and safety of humans	3.15 ^a^ (0.11)	3.16 ^a^ (0.11)	2.74 ^b^ (0.13)
These pet reptiles pose risk for health and safety of other animals	2.91 (0.11)	3.05 (0.11)	2.76 (0.13)
These pet reptiles pose risk for environment	2.45 (0.11)	2.50 (0.10)	2.38 (0.13)

^A^—answered before attending the course on animal welfare; ^B^—answered after the course; * 1—totally disagree; 5—totally agree; ^a,b,c^—values in the same row marked with different letters differ significantly (*p* < 0.05).

**Table 5:** Mean responses of first-year and sixth year veterinary students on their self-reported knowledge about pet reptile feeding, housing, health and behaviour.

Statement	Study Year		
	First ^A^ (*n* = 130)	First ^B^ (*n* = 123)	Sixth (*n* = 95)
	Mean * (SEM)		
I have sufficient knowledge about their feeding	2.50 (0.11)	2.59 (0.11)	2.50 (0.12)
I have sufficient knowledge about their housing	2.44 (0.10)	2.53 (0.09)	2.50 (0.12)
I have sufficient knowledge about their health	2.12 a (0.09)	2.45 b (0.10)	2.37 (0.12)
I have sufficient knowledge about their behaviour	2.29 (0.10)	2.42 (0.09)	2.44 (0.11)

^A^—answered before attending the course on animal welfare; ^B^—answered after the course; * 1—totally disagree; 5—totally agree; ^a,b^—values in the same row marked with different letters differ significantly (*p* < 0.05).

## Data Availability

The original contributions presented in the study are included in the article and Supplementary Materials; further inquiries can be addressed to the corresponding author.

## Supplementary Materials

The following are available online at https://www.mdpi.com/article/10.3390/ani11113185/s1, Questionnaire questions/statements as presented in the article, Table S1: Mean responses of veterinary students to statements related to pet turtles according to study years, Table S2: Mean responses of veterinary students to statements related to pet lizards according to study years, Table S3: Mean responses of veterinary students to statements related to pet snakes according to study years, Table S4: Mean responses of veterinary students on their self-reported knowledge about pet reptile feeding, housing, health and behaviour according to study years.

## References

[1] Ryan S., Bacon H., Endenburg N., Hazel S., Jouppi R., Lee N., Seksel K., Takashima G. (2019). WSAVA animal welfare guidelines for companion animal practitioners and veterinary teams. J. Small Anim. Pract..

[2] Ostović M., Mesić Ž., Mikuš T., Matković K., Pavičić Ž. (2016). Attitudes of veterinary students in Croatia toward farm animal welfare. Anim. Welf..

[3] Mikuš T., Ostović M., Sabolek I., Matković K., Pavičić Ž., Mikuš O., Mesić Ž. (2020). Opinions towards companion animals and their welfare: A survey of Croatian veterinary students. Animals.

[4] Levine E.D., Mills D.S., Houpt K.A. (2005). Attitudes of veterinary students at one US college toward factors relating to farm animal welfare. J. Vet. Med. Educ..

[5] Magnani D., Ferri N., Dalmau A., Messori S. (2017). Knowledge and opinions of veterinary students in Italy toward animal welfare science and law. Vet. Rec..

[6] Pirrone F., Mariti C., Gazzano A., Albertini M., Sighieri C., Diverio S. (2019). Attitudes toward animals and their welfare among Italian veterinary students. Vet. Sci..

[7] Mayer J., Martin J. (2005). Barriers to exotic animal medicine. Vet. Clin. N. Am. Exot. Anim. Pract..

[8] Siğirci B.D., Ikiz S., Çelik B., Ak S. (2019). A survey study on self-evaluations of small pet practitioners about exotic pets in Istanbul in 2016. Acta Vet. Eurasia.

[9] Williams D.L., Jackson R. (2016). Availability of information on reptile health and welfare from stores selling reptiles. Open J. Vet. Med..

[10] Warwick C., Steedman C., Jessop M., Toland E., Lindley S. (2014). Assigning degrees of ease or difficulty for pet animal maintenance: The EMODE system concept. J. Agric. Environ. Ethics.

[11] Warwick C., Steedman C., Jessop M., Arena P., Pilny A., Nicholas E. (2018). Exotic pet suitability: Understanding some problems and using a labeling system to aid animal welfare, environment, and consumer protection. J. Vet. Behav..

[12] Janovcová M., Rádlová S., Polák J., Sedláčková K., Peléšková Š., Žampachová B., Frynta D., Landová E. (2019). Human attitude toward reptiles: A relationship between fear, disgust, and aesthetic preferences. Animals.

[13] Learmonth M.J. (2020). The matter of non-avian reptile sentience, and why it “matters” to them: A conceptual, ethical and scientific review. Animals.

[14] Statista Number of Pet Animals in Europe in 2020, by Animal Type (in 1000s). https://www.statista.com/statistics/453880/pet-population-europe-by-animal/.

[15] Robinson J.E., St. John F.A.V., Griffiths R.A., Roberts D.L. (2015). Captive reptile mortality rates in the home and implications for the wildlife trade. PLoS ONE.

[16] Pasmans F., Bogaerts S., Braeckman J., Cunningham A.A., Hellebuyck T., Griffiths R.A., Sparreboom M., Schmidt B.R., Martel A. (2017). Future of keeping pet reptiles and amphibians: Towards integrating animal welfare, human health and environmental sustainability. Vet. Rec..

[17] Krysko K.L., Somma L.A., Smith D.C., Gillette C.R., Cueva D., Wasilewski J.A., Enge K.M., Johnson S.A., Campbell T.S., Edwards J.R. (2016). New verified nonindigenous amphibians and reptiles in Florida through 2015, with a summary of over 152 years of introductions. Reptiles Amphib..

[18] Toland E., Warwick C., Arena P. (2012). Pet hate: Exotic pet-keeping is on the rise despite decades of initiatives aimed at reducing the trade of exotic and rare animals. Three experts argue that urgent action is needed to protect both animals and ecosystems. Biologist.

[19] Grant R.A., Montrose V.T., Wills A.P. (2017). ExNOTic: Should we be keeping exotic pets?. Animals.

[20] Hess L. (2011). Exotic animals: Appropriately owned pets or inappropriately kept problems?. J. Avian Med. Surg..

[21] Whitehead M., Wilkinson A., Hoehfurtner T., Burman O., Collins L. UK vets’ opinions on keeping reptiles as pets. Proceedings of the BSAVA Congress.

[22] Proctor H. (2012). Animal sentience: Where are we and where are we heading?. Animals.

[23] National Research Council (NRC) Committee on Recognition and Alleviation of Pain in Laboratory Animals (2009). Recognition and Alleviation of Pain in Laboratory Animals.

[24] Malik A. (2018). Pain in reptiles: A review for veterinary nurses. Vet. Nurs. J..

[25] Benn A.L., McLelland D.J., Whittaker A.L. (2019). A review of welfare assessment methods in reptiles, and preliminary application of the Welfare Quality^®^ Protocol to the pygmy blue-tongue skink, *Tiliqua adelaidensis*, using animal-based measures. Animals.

[26] Lambert H., Carder G., D’Cruze N. (2019). Given the cold shoulder: A review of the scientific literature for evidence of reptile sentience. Animals.

[27] Broom D.M. (2016). Considering animals’ feelings: Précis of sentience and animal welfare (Broom 2014). Anim. Sentience.

[28] Schuppli C.A., Fraser D., Bacon H.J. (2014). Welfare of non-traditional pets. Rev. Sci. Tech. Off. Int. Epiz..

[29] Warwick C., Arena P., Steedman C. (2019). Spatial considerations for captive snakes. J. Vet. Behav..

[30] Sabolek I., Matković K., Pavičić Ž., Nejedli S., Piplica A., Ostović M. Risks to environment and native animals posed by exotic alien species. Proceedings of the 2nd International Scientific and Professional Meeting on Reptiles and Exotic Animals “REPTILIA”.

[31] McCleery R.A., Sovie A., Reed R.N., Cunningham M.W., Hunter M.E., Hart K.M. (2015). Marsh rabbit mortalities tie pythons to the precipitous decline of mammals in the Everglades. Proc. R. Soc. B.

[32] European Commission (EC) (2017). Invasive Alien Species of Union Concern.

[33] Boršić I., Ješovnik A., Mihinjač T., Kutleša P., Slivar S., Cigrovski Mustafić M., Desnica S. (2018). Invasive alien species of Union concern (Regulation 1143/2014) in Croatia. Nat. Croat..

[34] Warwick C., Steedman C. (2012). Injuries, envenomations and stings from exotic pets. J. R. Soc. Med..

[35] Mermin J., Hutwagner L., Vugia D., Shallow S., Daily P., Bender J., Koehler J., Marcus R., Angulo F.J. (2004). Reptiles, amphibians, and human *Salmonella* infection: A population-based, case-control study. Clin. Infect. Dis..

[36] Murphy D., Oshin F. (2015). Reptile-associated salmonellosis in children aged under 5 years in South West England. Arch. Dis. Child..

[37] Pees M., Rabsch W., Plenz B., Fruth A., Prager R., Simon S., Schmidt V., Münch S., Braun P.G. (2013). Evidence for the transmission of *Salmonella* from reptiles to children in Germany, July 2010 to October 2011. Euro Surveill..

[38] Lukac M., Pedersen K., Prukner-Radovcic E. (2015). Prevalence of *Salmonella* in captive reptiles from Croatia. J. Zoo Wildl. Med..

[39] Corrente M., Sangiorgio G., Grandolfo E., Bodnar L., Catella C., Trotta A., Martella V., Buonavoglia D. (2017). Risk for zoonotic *Salmonella* transmission from pet reptiles: A survey on knowledge, attitudes and practices of reptile-owners related to reptile husbandry. Prev. Vet. Med..

[40] Vučinić M., Hajzler I., Terzin J., Nenadović K., Janković L.J., Voslarova E., Vučićević M. (2019). Reptile ownership in Balkan countries: Demographics and reliance on veterinary advice. Anthrozoös.

