# Supplementary material for: A Survey Study of Veterinary Student Opinions and Knowledge about Pet Reptiles and Their Welfare
Source: Animals (Basel). 2021 Nov 8;11(11):3185. doi: 10.3390/ani11113185 (PMC8614325; doi:10.3390/ani11113185)
Supplement: Supplementary file 1 [file animals-11-03185-s001.zip › animals-1424675-supplementary.pdf]

Supplementary Materials

Questionnaire questions/statements as presented in the article.

1. Study year: 1 2 3 4 5 6
2. Preferred/chosen study track: a) Pet animals b) Farm animals and horses c) Hygiene and technology of animal foodstuffs and veterinary public health d) I do not know
3. Gender: a) Male b) Female
4. Age (years): ___________
5. Early environment: a) Rural b) Urban
6. Secondary education: a) High school b) Veterinary school c) Other ___________
7. Have you owned or kept pet animals? a) Yes b) No
8. Have you owned or kept exotic pet animals (i.e., any species other than dogs and cats)? a) Yes b) No
9. Please indicate your level of agreement/disagreement (5 – totally agree, 4 – agree, 3 – neutral/unsure, 2 – disagree, 1 – totally disagree) with the statement that the following reptiles are capable of thinking:

Turtles (Chelonians)	5	4	3	2	1
Lizards	5	4	3	2	1
Snakes	5	4	3	2	1

10. Please indicate your level of agreement/disagreement (5 – totally agree, 4 – agree, 3 – neutral/unsure, 2 – disagree, 1 – totally disagree) with the statement that the following reptiles are capable of feeling emotions:

Turtles (Chelonians)	5	4	3	2	1
Lizards	5	4	3	2	1
Snakes	5	4	3	2	1

11. Please indicate your level of agreement/disagreement (5 – totally agree, 4 – agree, 3 – neutral/unsure, 2 – disagree, 1 – totally disagree) with the following statements related to pet reptiles:

	Turtles (Chelonians)	Lizards	Snakes
Biological functioning is important for their welfare			
Emotional states are important for their welfare			
Natural living is important for their welfare			

12. Please indicate your level of agreement/disagreement (5 – totally agree, 4 – agree, 3 – neutral/unsure, 2 – disagree, 1 – totally disagree) with the statement that keeping the following reptiles as pets is acceptable:

Turtles (Chelonians)	5	4	3	2	1
Lizards	5	4	3	2	1
Snakes	5	4	3	2	1

13. Please indicate your level of agreement/disagreement (5 – totally agree, 4 – agree, 3 – neutral/unsure, 2 – disagree, 1 – totally disagree) with the statement that owners are properly informed about the following pet reptiles and their needs prior to acquisition:

Turtles (Chelonians)	5	4	3	2	1
Lizards	5	4	3	2	1
Snakes	5	4	3	2	1

14. Please indicate your level of agreement/disagreement (5 – totally agree, 4 – agree, 3 – neutral/unsure, 2 – disagree, 1 – totally disagree) with the statement that welfare of the following pet reptiles is compromised:

Turtles (Chelonians)	5	4	3	2	1
Lizards	5	4	3	2	1
Snakes	5	4	3	2	1

15. Please indicate your level of agreement/disagreement (5 – totally agree, 4 – agree, 3 – neutral/unsure, 2 – disagree, 1 – totally disagree) with the statements that the following pet reptiles pose risk for:

	Turtles (Chelonians)	Lizards	Snakes
Health and safety of humans			
Health and safety of other animals			
Environment			

16. Please indicate your level of agreement/disagreement (5 – totally agree, 4 – agree, 3 – neutral/unsure, 2 – disagree, 1 – totally disagree) with the following statements related to pet reptiles:

I have sufficient knowledge about their feeding	5	4	3	2	1
I have sufficient knowledge about their housing	5	4	3	2	1
I have sufficient knowledge about their health	5	4	3	2	1
I have sufficient knowledge about their behaviour	5	4	3	2	1

Table S1. Mean responses of veterinary students to statements related to pet turtles according to study years.

Statement	Study Year						
	First [A] (n = 130)	First [B] (n = 123)	Second (n = 108)	Third (n = 105)	Fourth (n = 90)	Fifth (n = 68)	Sixth (n = 95)
	Mean * (SEM)						
These reptiles are capable of thinking	2.82 (0.07)	3.24 (0.08)	3.12 (0.08)	2.96 (0.09)	2.96 (0.11)	3.41 (0.12)	3.04 (0.10)
These reptiles are capable of feeling emotions	2.65 (0.09)	3.11 (0.08)	3.19 (0.10)	2.80 (0.11)	3.02 (0.12)	3.40 (0.12)	2.91 (0.11)
Biological functioning is important for their welfare	4.44 (0.08)	4.61 (0.06)	4.71 (0.08)	4.83 (0.06)	4.82 (0.07)	4.46 (0.13)	4.78 (0.07)
Emotional states are important for their welfare	3.22 (0.10)	3.84 (0.09)	3.89 (0.12)	3.91 (0.11)	3.51 (0.13)	3.79 (0.16)	3.48 (0.14)
Natural living is important for their welfare	3.70 (0.10)	4.21 (0.10)	4.69 (0.08)	4.79 (0.07)	4.73 (0.07)	4.38 (0.14)	4.71 (0.08)
It is acceptable to keep these reptiles as pets	3.09 (0.11)	3.13 (0.11)	3.14 (0.11)	3.16 (0.12)	3.19 (0.12)	3.13 (0.14)	3.33 (0.12)
Owners are properly informed prior to acquisition	3.39 (0.12)	2.99 (0.11)	3.05 (0.13)	3.13 (0.13)	3.21 (0.15)	2.87 (0.13)	3.06 (0.13)
The welfare of these pet reptiles is compromised	3.37 (0.09)	3.47 (0.08)	3.55 (0.10)	3.51 (0.10)	3.70 (0.11)	3.22 (0.13)	3.47 (0.10)
These pet reptiles pose risk for health and safety of humans	1.86 (0.08)	2.24 (0.10)	1.85 (0.09)	1.98 (0.11)	1.91 (0.10)	2.16 (0.12)	2.37 (0.12)
These pet reptiles pose risk for health and safety of other animals	1.99 (0.09)	2.45 (0.11)	1.91 (0.10)	1.82 (0.10)	1.88 (0.10)	2.16 (0.12)	2.45 (0.13)
These pet reptiles pose risk for environment	1.99 (0.09)	2.43 (0.11)	1.98 (0.11)	1.90 (0.12)	2.01 (0.11)	2.22 (0.14)	2.28 (0.13)

A – answered before attending the course on animal welfare; B – answered after the course; * 1 – totally disagree; 5 – totally agree.

Table S2. Mean responses of veterinary students to statements related to pet lizards according to study years.

Statement	Study Year						
	First [A] (n = 130)	First [B] (n = 123)	Second (n = 108)	Third (n = 105)	Fourth (n = 90)	Fifth (n = 68)	Sixth (n = 95)
	Mean * (SEM)						
These reptiles are capable of thinking	2.72 (0.08)	3.17 (0.09)	3.03 (0.09)	2.97 (0.08)	2.84 (0.11)	3.38 (0.11)	2.90 (0.10)
These reptiles are capable of feeling emotions	2.37 (0.09)	2.87 (0.08)	2.95 (0.11)	2.62 (0.11)	2.68 (0.12)	3.28 (0.13)	2.57 (0.11)
Biological functioning is important for their welfare	4.28 (0.10)	4.61 (0.07)	4.72 (0.07)	4.81 (0.06)	4.80 (0.07)	4.41 (0.14)	4.75 (0.07)
Emotional states are important for their welfare	2.91 (0.10)	3.44 (0.12)	3.83 (0.12)	3.84 (0.11)	3.40 (0.14)	3.62 (0.16)	3.33 (0.14)
Natural living is important for their welfare	3.52 (0.11)	4.25 (0.10)	4.69 (0.08)	4.76 (0.07)	4.72 (0.08)	4.34 (0.14)	4.68 (0.08)
It is acceptable to keep these reptiles as pets	2.82 (0.11)	2.92 (0.11)	3.09 (0.12)	3.25 (0.12)	3.16 (0.12)	3.13 (0.14)	3.22 (0.13)
Owners are properly informed prior to acquisition	3.59 (0.12)	3.08 (0.12)	3.17 (0.13)	3.19 (0.13)	3.31 (0.14)	2.90 (0.13)	3.17 (0.13)
The welfare of these pet reptiles is compromised	3.28 (0.08)	3.61 (0.09)	3.60 (0.11)	3.51 (0.11)	3.60 (0.10)	3.22 (0.13)	3.40 (0.11)
These pet reptiles pose risk for health and safety of humans	2.23 (0.09)	2.41 (0.09)	2.20 (0.10)	2.32 (0.11)	2.11 (0.11)	2.28 (0.14)	2.44 (0.12)
These pet reptiles pose risk for health and safety of other animals	2.31 (0.09)	2.51 (0.10)	2.30 (0.12)	2.13 (0.12)	2.18 (0.11)	2.27 (0.13)	2.53 (0.13)
These pet reptiles pose risk for environment	2.15 (0.10)	2.16 (0.09)	2.11 (0.11)	1.98 (0.12)	2.00 (0.11)	2.25 (0.14)	2.33 (0.13)

A – answered before attending the course on animal welfare; B – answered after the course; * 1 – totally disagree; 5 – totally agree.

Table S3. Mean responses of veterinary students to statements related to pet snakes according to study years.

Statement	Study Year						
	First [A] (n = 130)	First [B] (n = 123)	Second (n = 108)	Third (n = 105)	Fourth (n = 90)	Fifth (n = 68)	Sixth (n = 95)
	Mean * (SEM)						
These reptiles are capable of thinking	2.88 (0.09)	3.11 (0.09)	3.11 (0.10)	3.00 (0.09)	2.86 (0.11)	3.40 (0.12)	2.80 (0.11)
These reptiles are capable of feeling emotions	2.31 (0.09)	2.83 (0.10)	3.01 (0.12)	2.71 (0.12)	2.66 (0.13)	3.37 (0.13)	2.52 (0.11)
Biological functioning is important for their welfare	4.23 (0.10)	4.52 (0.08)	4.75 (0.07)	4.82 (0.05)	4.81 (0.07)	4.40 (0.14)	4.71 (0.08)
Emotional states are important for their welfare	2.75 (0.11)	3.32 (0.12)	3.72 (0.13)	3.71 (0.13)	3.36 (0.15)	3.53 (0.17)	3.31 (0.14)
Natural living is important for their welfare	3.66 (0.11)	4.13 (0.11)	4.65 (0.08)	4.74 (0.08)	4.70 (0.08)	4.29 (0.15)	4.66 (0.08)
It is acceptable to keep these reptiles as pets	2.70 (0.11)	2.69 (0.11)	2.90 (0.11)	3.16 (0.12)	3.07 (0.12)	2.99 (0.13)	3.11 (0.13)
Owners are properly informed prior to acquisition	3.59 (0.12)	2.99 (0.12)	3.19 (0.13)	3.28 (0.14)	3.38 (0.14)	2.96 (0.14)	3.22 (0.14)
The welfare of these pet reptiles is compromised	3.38 (0.09)	3.57 (0.09)	3.61 (0.11)	3.63 (0.11)	3.53 (0.11)	3.22 (0.13)	3.38 (0.11)
These pet reptiles pose risk for health and safety of humans	3.15 (0.11)	3.16 (0.11)	2.74 (0.12)	2.90 (0.11)	2.70 (0.13)	2.57 (0.14)	2.74 (0.13)
These pet reptiles pose risk for health and safety of other animals	2.91 (0.11)	3.05 (0.11)	2.81 (0.13)	2.61 (0.12)	2.64 (0.13)	2.56 (0.14)	2.76 (0.13)
These pet reptiles pose risk for environment	2.45 (0.11)	2.50 (0.10)	2.24 (0.12)	2.10 (0.12)	2.23 (0.12)	2.43 (0.14)	2.38 (0.13)

A – answered before attending the course on animal welfare; B – answered after the course; * 1 – totally disagree; 5 – totally agree.

Table S4. Mean responses of veterinary students on their self-reported knowledge about pet reptile feeding, housing, health and behaviour according to study years.

Statement	Study Year						
	First [A] (n = 130)	First [B] (n = 123)	Second (n = 108)	Third (n = 105)	Fourth (n = 90)	Fifth (n = 68)	Sixth (n = 95)
	Mean * (SEM)						
I have sufficient knowledge about their feeding	2.50 (0.11)	2.59 (0.11)	2.45 (0.11)	2.51 (0.11)	2.49 (0.11)	2.37 (0.11)	2.50 (0.12)
I have sufficient knowledge about their housing	2.44 (0.10)	2.53 (0.09)	2.54 (0.11)	2.66 (0.11)	2.46 (0.11)	2.44 (0.11)	2.50 (0.12)
I have sufficient knowledge about their health	2.12 (0.09)	2.45 (0.10)	2.22 (0.09)	2.32 (0.09)	2.44 (0.11)	2.35 (0.10)	2.37 (0.12)
I have sufficient knowledge about their behaviour	2.29 (0.10)	2.42 (0.09)	2.46 (0.11)	2.70 (0.11)	2.54 (0.11)	2.41 (0.11)	2.44 (0.11)

A – answered before attending the course on animal welfare; B – answered after the course; * 1 – totally disagree; 5 – totally agree.
